# *Ginkgo biloba* extract ameliorates skin fibrosis in a bleomycin-induced mouse model of systemic sclerosis

**DOI:** 10.1080/19768354.2024.2337761

**Published:** 2024-04-18

**Authors:** Beomgu Lee, Jong Seong Roh, Hoim Jeong, Yerin Kim, Jihyeon Lee, Changun Yun, Jiyoung Park, Da-sol Kim, Jungsoo Lee, Min Wook So, Aran Kim, Dong Hyun Sohn, Seung-Geun Lee

**Affiliations:** a Department of Microbiology and Immunology, Pusan National University School of Medicine, Yangsan, Republic of Korea; b Department of Herbal Prescription, College of Korean Medicine, Daegu Haany University, Gyeongsan, Republic of Korea; c Department of Biological Sciences, College of Information and Biotechnology, Ulsan National Institute of Science and Technology, Ulsan, Republic of Korea; d Department of Dermatology, Pusan National University Yangsan Hospital, Yangsan, Republic of Korea; e Research Institute for Convergence of Biomedical Science and Technology, Pusan National University Yangsan Hospital, Yangsan, Republic of Korea; f Division of Rheumatology, Department of Internal Medicine, Pusan National University School of Medicine, Pusan National University Yangsan Hospital, Yangsan, Republic of Korea; g Division of Rheumatology, Department of Internal Medicine, Pusan National University School of Medicine, Pusan National University Hospital, Busan, Republic of Korea; h Biomedical Research Institute, Pusan National University Hospital, Busan, Republic of Korea

**Keywords:** Systemic sclerosis (SSc), *Ginkgo biloba* extract (GBE), transforming growth factor (TGF)-β, skin fibroblast, adipocyte-myofibroblast transition (AMT)

## Abstract

Systemic sclerosis (SSc) is a chronic autoimmune disease characterized by skin and internal organ fibrosis and obliterative vasculopathy. Few effective treatments are currently available for fibrosis in SSc, therefore, demand persists for novel therapies. Although use of *Ginkgo biloba* extract (GBE) has been reported to improve blood circulation and alleviate liver and lung fibrosis, its effect on skin fibrosis in SSc remains unclear. In this study, the effects and underlying mechanisms of GBE on skin fibrosis in bleomycin (BLM)-induced mouse model of SSc was investigated. GBE significantly reduced dermal thickness and protein levels of profibrotic factors in the BLM-induced SSc mouse model. Moreover, GBE inhibited the gene expression of profibrotic factors, such as COL1A1, α-SMA, and connective tissue growth factor (CTGF), in fibroblasts by suppressing transforming growth factor (TGF)-β signaling. Furthermore, GBE inhibited the transdifferentiation of adipocytes into myofibroblasts. Thus, our findings suggest that GBE is a promising therapeutic candidate for the treatment of SSc.

## Introduction

Systemic sclerosis (SSc) is a chronic autoimmune disease characterized by excessive fibrosis that affects the skin and multiple organs (Brown and O’Reilly 2019). Fibrosis involves the abnormal deposition of collagen and other extracellular matrix components (Jimenez and Derk 2004; Gabrielli et al. 2009). Despite the high morbidity and mortality rates of SSc among immune-mediated rheumatic diseases, therapeutic options for treatment of SSc are limited (Moon et al. 2018), highlighting the need for novel approaches to ameliorate fibrosis in SSc.

Among the experimental animal models of SSc, the bleomycin (BLM)-induced SSc model is commonly used to study its pathogenesis (Roh et al. 2021). BLM increases the mRNA expression of extracellular matrix proteins such as type I collagen, fibronectin, and decorin in human dermal fibroblasts (Clark et al. 1980; Yamamoto et al. 2000). Moreover, BLM induces the expression of transforming growth factor (TGF)-β and connective tissue growth factor (CTGF) in skin fibroblasts (Yamamoto et al. 2000). Both are fibrogenic cytokines that play crucial roles in tissue fibrosis (Ihn 2002; Wang et al. 2011). In particular, TGF-β stimulates the proliferation and transition of fibroblasts into myofibroblasts, and collagen deposition during fibrosis through both the canonical Smad2/3 activation and non-Smad signaling pathways including the AKT and Wnt/β-catenin (Akhmetshina et al. 2012; Finnson et al. 2020; Li et al. 2023).

Although myofibroblasts play a key role in skin fibrosis in SSc, their origin in fibrotic tissues remains controversial (Ebmeier and Horsley 2015; Korman 2019). In addition to the activation of resident fibroblasts by TGF-β and other profibrotic stimuli, myofibroblasts can be differentiated from epithelial cells and endothelial cells via epithelial-mesenchymal transition (EMT) and endothelial-mesenchymal transition (EndoMT), respectively (Piera-Velazquez et al. 2011; Korman 2019). Monocyte-derived fibrocytes also have the potential to differentiate into myofibroblasts and accumulate in the lung tissues of patients with SSc (Tourkina et al. 2011; Korman 2019). Adipocytes are another important source of myofibroblasts in patients with SSc. Loss of intradermal adipose tissue and its replacement with fibrous tissue were observed in mice with BLM-induced fibrosis through adipocyte-myofibroblast transition (AMT) (Marangoni et al. 2015; Varga and Marangoni 2017).

*Ginkgo biloba* extract (GBE), which is derived from the leaves of the *Ginkgo biloba* tree, has been recognized for its therapeutic properties against various disorders, including atherosclerotic cardiovascular diseases, diabetic retinopathy, dementia, delirium, and tinnitus (Ji et al. 2023). GBE contains many constituents, with flavonoids and terpenoids as the main active components, which can exert antioxidant, antiplatelet, and anti-inflammatory effects (Liu et al. 2021; Ji et al. 2023). However, the exact underlying mechanisms remain unclear. GBE has also been reported to alleviate the fibrosis of liver, lung, and kidney (Iraz et al. 2006; Al-Attar 2012; Lu et al. 2015; Wang et al. 2015). However, the effect of GBE on skin fibrosis in patients with SSc remains unclear. In this study, the protective effects of GBE on SSc in both *in vivo* and *in vitro* models using BLM-induced mouse model of SSc and TGF-β-induced fibrosis were investigated.

## Materials and Methods

### Reagents

Bleomycin (BLM) and *Ginkgo biloba* extract (GBE) were donated by Dong-A ST Co., Ltd. and SK Chemicals Co., Ltd., respectively. Recombinant human and mouse TGF-β1 proteins were purchased from R&D Systems.

### SSc mouse model

All animal experiments were approved by the Institutional Animal Care and Use Committee of Pusan National University (PNU-2023-0385), as previously described (Roh et al. 2021). Briefly, 8 weeks old male BALB/c mice were randomly assigned to four groups: phosphate-buffered saline (PBS), BLM, BLM + GBE (100 mg/kg), and BLM + GBE (200 mg/kg). BLM was dissolved in 1× sterile Dulbecco’s phosphate-buffered saline (DPBS) at a concentration of 1 mg/ml and GBE was dissolved in sterile distilled water (D.W.). For the BLM and GBE groups, 100 µl of BLM was subcutaneously injected into the shaved back of mice on alternate days for three weeks, while the PBS group was injected with the same volume of 1× DPBS. GBE (100 or 200 mg/kg/day) was orally administered with the BLM injection for three weeks, whereas the PBS and BLM groups received the same volume of D.W.

### Histological analysis

To measure the thickness of the dermal layer, skin tissues were processed into paraffin sections and stained with hematoxylin and eosin (H&E). The deposition of collagen fibers was visualized using Masson’s trichrome staining as previously described (Yun et al. 2023). The dermal layer was defined as the area from the dermal-epidermal junction to the subcutaneous fat junction. Two independent examiners measured the thicknesses of five randomly selected areas per specimen. Images were captured using a microscope (Olympus BX51) and quantified using iSolution Lite 9.1 (Image & Microscope Technology).

### Fibroblast culture

Written informed consent was obtained from all donors and the experimental protocol was approved by the Institutional Review Board of Pusan National University Yangsan Hospital (11-2023-013). To obtain normal human dermal fibroblasts, skin tissues were washed twice with 1× DPBS containing 200 units/ml penicillin–streptomycin (Thermo Fisher Scientific). The epidermis was removed and the dermal portion was finely diced into approximately 1 mm × 1 mm × 1 mm pieces using scissors. The pieces were carefully placed on a 150 mm cell culture plate and Dulbecco’s modified Eagle’s medium (DMEM) (Sigma-Aldrich) supplemented with 10% fetal bovine serum (FBS), 100 units/ml of penicillin, and 100 µg/ml of streptomycin (Thermo Fisher Scientific) was added. The cell culture plate was incubated at 37 °C in a 5% CO_2_ atmosphere until dermal fibroblasts formed out of the tissue. When the plate reached approximately 70% confluence, the initial subculture was performed. Mouse embryonic fibroblasts NIH3T3 were cultured in DMEM supplemented with 10% bovine serum (BS), 100 units/ml of penicillin, and 100 µg/ml of streptomycin. NIH3T3 (3 × 10^5^ cells/well) or normal human dermal fibroblasts (3 × 10^5^ cells/well) were placed into 6-well culture plates a day before treatment with TGF-β1 and GBE. On the day of treatment, NIH3T3 were treated with mouse TGF-β1 (10 ng/ml) in DMEM (0.1% BS) and normal human dermal fibroblasts were treated with human TGF-β1 (10 ng/ml) in serum-free DMEM for 24 h. GBE, dissolved in 20% dimethyl sulfoxide (100 mg/ml), was treated at final concentrations ranging from 0 to 200 µg/ml. After 24 h, cells were harvested for RNA or protein extraction.

### Adipocyte differentiation of 3T3-L1 and transdifferentiation into myofibroblasts

Adipocyte differentiation of 3T3-L1 was performed as previously described with minor modification (Eom et al. 2022). The 3T3-L1 mouse preadipocytes were seeded in 6-well plates at a density of 2 × 10^5^ cells/well and cultured in DMEM supplemented with 10% BS, 100 units/ml of penicillin, and 100 µg/ml of streptomycin for three days to reach confluence. To induce differentiation into adipocytes, the cells were incubated in DMEM supplemented with 10% FBS, 100 units/ml of penicillin, 100 µg/ml of streptomycin, and an adipogenic cocktail containing 500 μM 3-isobutyl-1-methylxanthine (IBMX), 1 μM dexamethasone, and 5 µg/ml insulin (Sigma-Aldrich) for two days. Subsequently, the cells were incubated in DMEM supplemented with 10% FBS, 100 units/ml of penicillin, 100 µg/ml of streptomycin, and 5 µg/ml insulin for six days, replacing the media every two days. To induce transdifferentiation into myofibroblasts, the differentiated 3T3-L1 was cultured in DMEM supplemented with 10% FBS, 100 units/ml of penicillin, and 100 µg/ml of streptomycin for 48 h in the presence of TGF-β1 (10 ng/ml). The control group was treated only with mouse TGF-β1 and the experimental groups were treated with both mouse TGF-β1 and GBE (0–200 µg/ml).

### Oil red O staining

Adipocyte differentiation and transdifferentiation into myofibroblasts were evaluated using Oil Red O staining. Cells were washed with 1× phosphate buffered saline (PBS) and fixed with 10% neutral buffered formalin for 1 h. The fixed cells were washed with D.W. and dehydrated with 60% isopropanol. After complete drying, the cells were stained with 5.1 mM Oil Red O solution (Sigma-Aldrich), which was filtered using a 0.22 µm filter for 10 min at room temperature. The Oil Red O solution was removed, the cells were washed with D.W., and images were captured using a microscope (Olympus CKX41). The relative lipid content stained with Oil Red O was quantified using ImageJ software.

### RNA isolation and quantitative real-time PCR

Total RNA was extracted using a RNeasy mini kit (QIAGEN) according to the manufacturer's protocol and reverse-transcribed into cDNA using a cDNA synthesis kit (SmartGene). Quantitative real-time PCR was performed using specific primers as previously described (Roh et al. 2021). The cycle threshold (Ct) values were normalized using both a housekeeping gene GAPDH and the control group (2^-ΔΔCT^ method) (Lee et al. 2022).

### Western blot analysis

Western blot analysis of cell lysates and skin tissue lysates was performed as previously described (Roh et al. 2021) using the following antibodies: anti-COL1A1 (3G3) (Santa Cruz Biotechnology), anti-phospho-Smad2/3 (D27F4) (#8828), anti-Smad2/3 (D7G7) (#8685), anti-phospho-AKT (ser473) (#4060), anti-phospho-AKT (thr308) (#2965), anti-AKT (pan) (#4691), anti-GAPDH (#2118) (Cell Signaling Technology), anti-β-actin (AC-15) (Sigma-Aldrich), and anti-α-SMA (MAB1420) (R&D Systems).

### Statistical analysis

Statistical analyses were performed using the GraphPad Prism software (GraphPad Software). Statistical significance was determined using the one-way analysis of variance (ANOVA), followed by Dunnett’s post hoc test. All data are presented as mean ± standard error of the mean (SEM) and *P* < 0.05 was considered statistically significant.

## Results

### Ginkgo biloba extract (GBE) ameliorated bleomycin (BLM)-induced skin fibrosis in vivo

**Figure 1.**
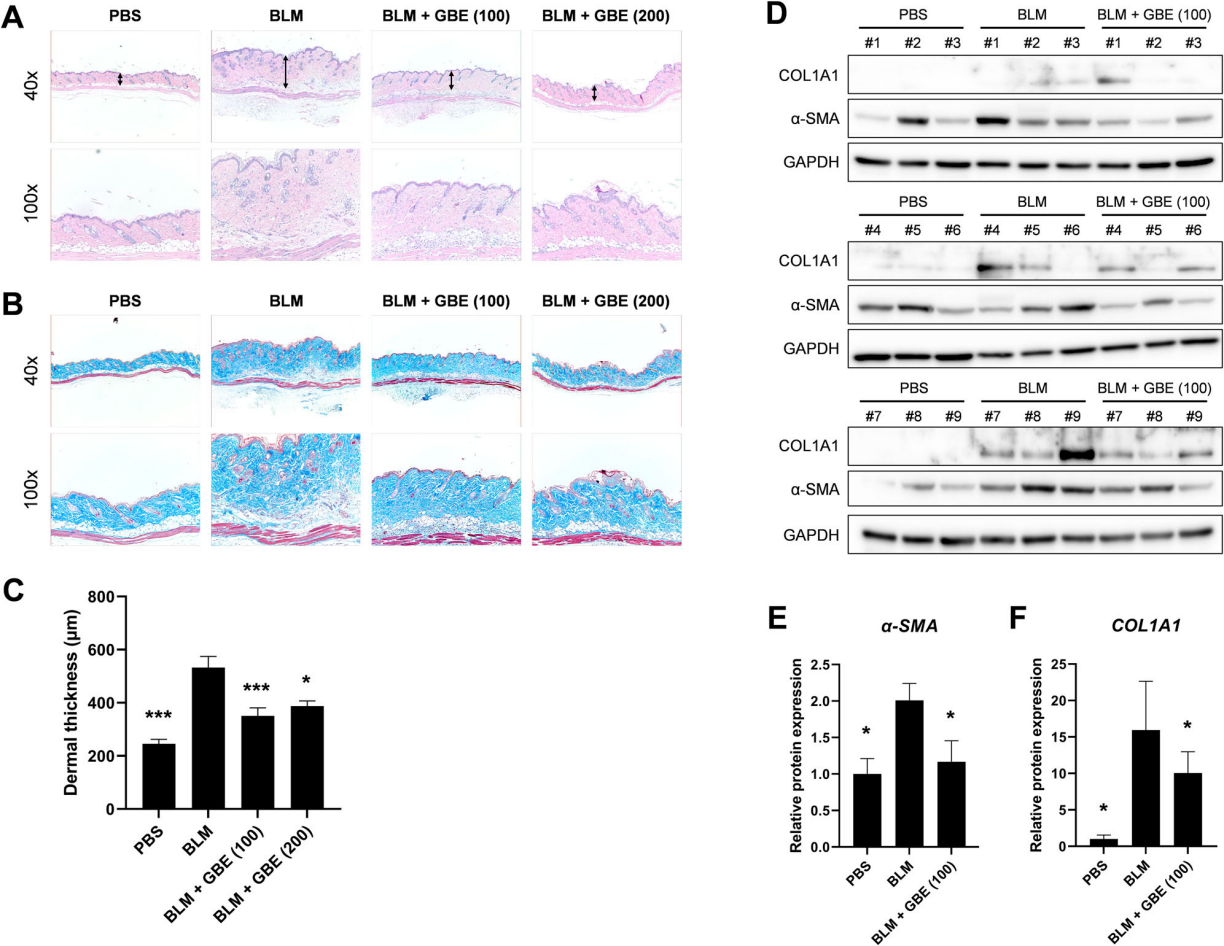
*Ginkgo biloba* extract (GBE) ameliorated skin fibrosis in bleomycin (BLM)-induced systemic sclerosis (SSc) mouse model. Mouse skin tissues were stained with hematoxylin and eosin (H&E) (A) and Masson’s trichrome (B) to visualize tissue morphology and collagen deposition, respectively. Original magnification: 40× or 100×. (C) The dermal thickness of each group (n = 9) was quantified. (D) The lesioned skins were analyzed by western blot to detect COL1A1 and α-SMA. GAPDH was used as loading control. BLM + GBE (100 mg/kg) group was used for the western blot analysis. (E and F) Band intensities from the western blot images were quantified by densitometry and the relative protein expression levels were compared with the PBS group. Data are presented as mean ± standard error of the mean (SEM). *P*-values were calculated using one-way ANOVA with Dunnett's post hoc test compared with the BLM group. *P < 0.05, ***P < 0.001. PBS, 1× DPBS injection group; BLM, Bleomycin injection group; BLM + GBE (100), Bleomycin injection and GBE (100 mg/kg) treatment group; BLM + GBE (200), Bleomycin injection and GBE (200 mg/kg) treatment group.

To explore the anti-fibrotic effect of GBE in systemic sclerosis (SSc), a BLM-induced skin fibrosis mouse model was used. Histological analyses revealed that the injection of BLM alone increased dermal thickness and collagen deposition compared to that of the PBS group (Figure 1(A) and 1(B)). On the contrary, oral administration of GBE (100 and 200 mg/kg) significantly reduced dermal thickness and collagen deposition compared to that of the BLM group (Figure 1(A) and 1(B)). The dermal thickness was found to be 246 µm ± 53 [PBS], 533 µm ± 41 [BLM], 351 µm ± 13 [GBE (100 mg/kg)], and 388 µm ± 63 [GBE (200 mg/kg)], respectively (Figure 1(C)). Moreover, the loss of intradermal adipose tissue and its replacement with fibrous tissue was observed following BLM injection, which was reduced by GBE treatment. Furthermore, histological changes correlated with the expression levels of the fibrosis markers COL1A1 and α-SMA. The protein expression of COL1A1 and α-SMA in the lesioned skin reduced in the groups treated with GBE compared to those in the BLM group (Figure 1(D)–(F)). These results suggest that GBE treatment ameliorated dermal fibrosis, probably by suppressing the expression of fibrosis markers and the transdifferentiation of adipocytes into myofibroblasts.

### GBE inhibited the expression of profibrotic factors induced by TGF-β

**Figure 2.**
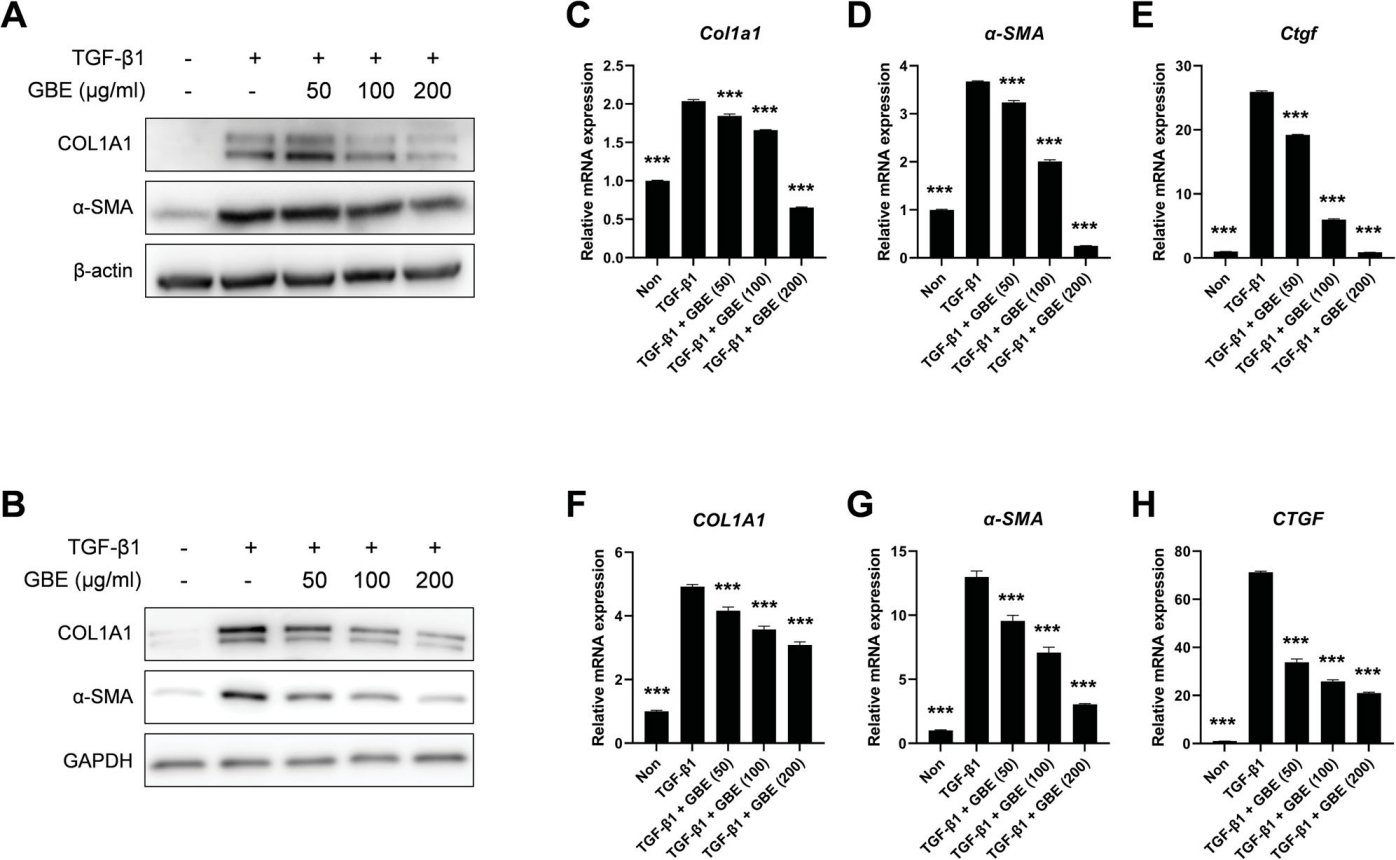
GBE downregulated the expression of fibrosis markers induced by TGF-β. (A and B) Mouse embryonic fibroblasts (NIH3T3) (A) and normal human dermal fibroblasts (B) were treated with TGF-β1 (10 ng/ml) and GBE for 24 h. The protein levels of COL1A1 and α-SMA were analyzed by western blot. Beta-actin or GAPDH was used as a loading control for western blot. (C–H) NIH3T3 cells (C–E) and normal human dermal fibroblasts (F–H) were treated with TGF-β1 (10 ng/ml) and GBE for 24 h and the relative mRNA expression levels of *COL1A1*, *α-SMA*, and *CTGF* were analyzed by qPCR. GAPDH was used as a normalization control for qPCR. Data are presented as mean ± SEM. *P*-values were calculated using one-way ANOVA with Dunnett's post hoc test compared with TGF-β1 group. ***P < 0.001.

To investigate the anti-fibrotic effect of GBE *in vitro*, the NIH3T3 mouse embryonic fibroblasts and normal human dermal fibroblasts were stimulated with recombinant TGF-β1 and the cells were treated with varying concentrations of GBE. The results showed that treatment with TGF-β1 significantly upregulated the protein levels of fibrosis markers, such as COL1A1 and α-SMA, which decreased on treatment with GBE in a dose-dependent manner (Figure 2(A) and 2(B)). We then examined the transcription levels of the fibrosis markers. As observed with respect to the protein levels, the mRNA levels of *COL1A1*, *α-SMA*, and *CTGF* induced by TGF-β1 exhibited a dose-dependent reduction upon treatment with GBE in both the NIH3T3 and normal human dermal fibroblasts (Figure 2(C)–(H)). These findings demonstrate that GBE reduces both mRNA and protein expression of fibrosis markers, suggesting its potential to ameliorate fibrosis induced by TGF-β.

### GBE inhibited transdifferentiation of adipocytes into myofibroblast

**Figure 3.**
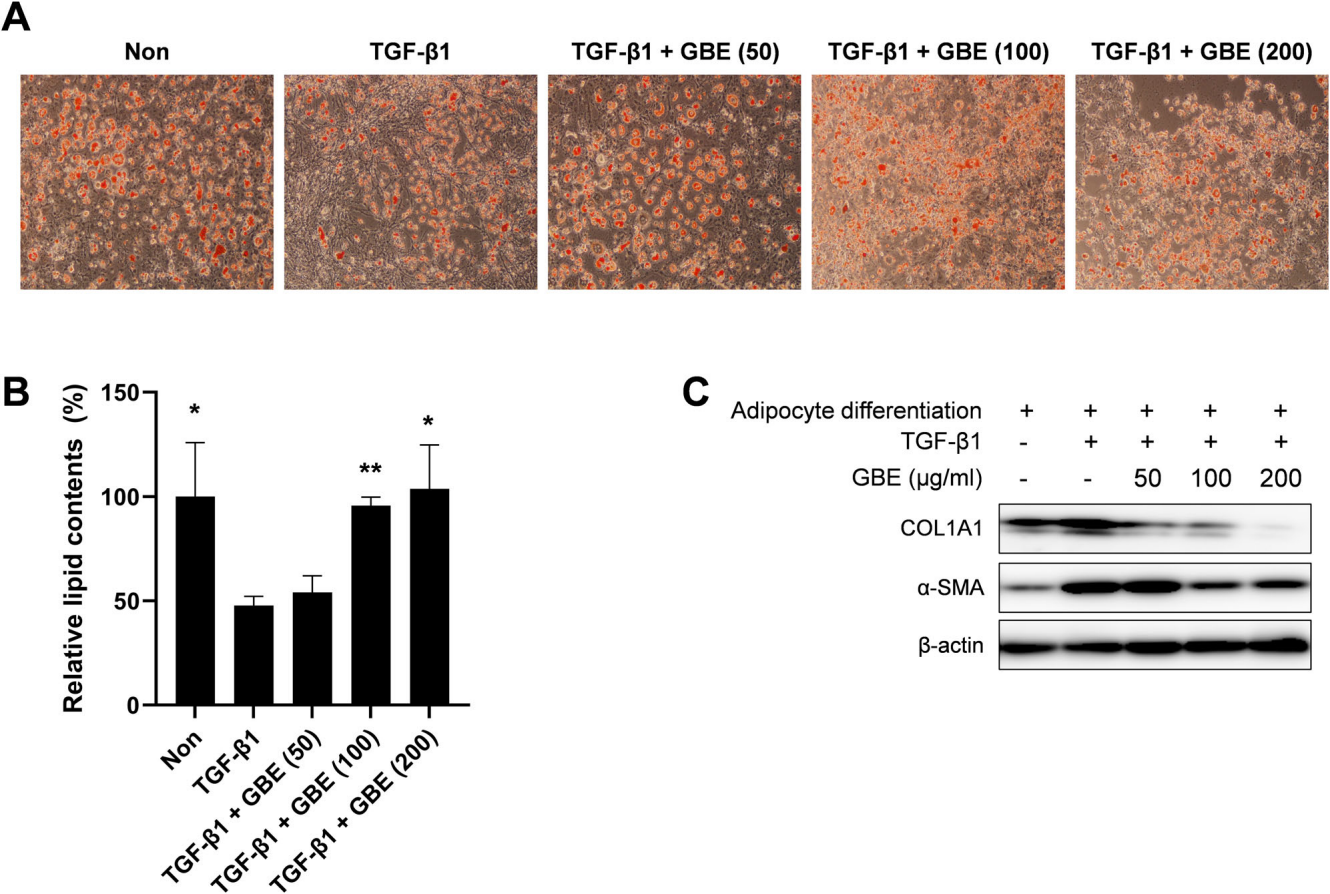
GBE inhibited transdifferentiation of adipocytes into myofibroblasts. (A–C) Murine preadipocytes, 3T3-L1, were cultured in adipogenic media for eight days to differentiate into adipocytes. The differentiated adipocytes were then transdifferentiated into myofibroblasts by treatment with TGF-β1 (10 ng/ml) for 48 h. Cells were also treated with GBE (50, 100, and 200 µg/ml). (A) Lipid was stained with Oil Red O and representative images were captured at ×100 magnification. (B) Relative lipid content from (A) was quantified using ImageJ. Data are presented as mean ± SEM. *P*-values were calculated using one-way ANOVA with Dunnett's post hoc test compared with the TGF-β1 group. *P < 0.05, **P < 0.01. (C) The protein levels of COL1A1 and α-SMA in the transdifferentiated myofibroblasts were analyzed by western blot. Beta-actin was used as a loading control.

Myofibroblasts within the fibrotic dermal tissue of SSc have been suggested to originate through the process of adipocyte-myofibroblast transition (AMT), resulting in the transformation of the intradermal adipose tissue into fibrous tissue in SSc (Marangoni et al. 2015; Varga and Marangoni 2017). Therefore, we investigated whether GBE modulated the transdifferentiation of adipocytes into myofibroblasts. The 3T3-L1 preadipocytes were differentiated into adipocytes in adipogenic media and then transdifferentiated into myofibroblast upon treatment with TGF-β1 in the presence of GBE. After eight days of incubation in adipogenic media, 3T3-L1 cells effectively differentiated into adipocytes, as indicated by their rounded shape and notable lipid staining with Oil Red O (Figure 3(A) and 3(B)). While the treatment with TGF-β1 led to changes in cell morphology and lipid content, GBE effectively suppressed these phenotypic changes (Figure 3(A) and 3(B)). Moreover, GBE demonstrated a dose-dependent inhibition of COL1A1 and α-SMA expression (Figure 3(C)). These results demonstrated that GBE inhibited adipocyte-myofibroblast transdifferentiation in response to TGF-β.

### GBE suppressed TGF-β signaling

**Figure 4.**
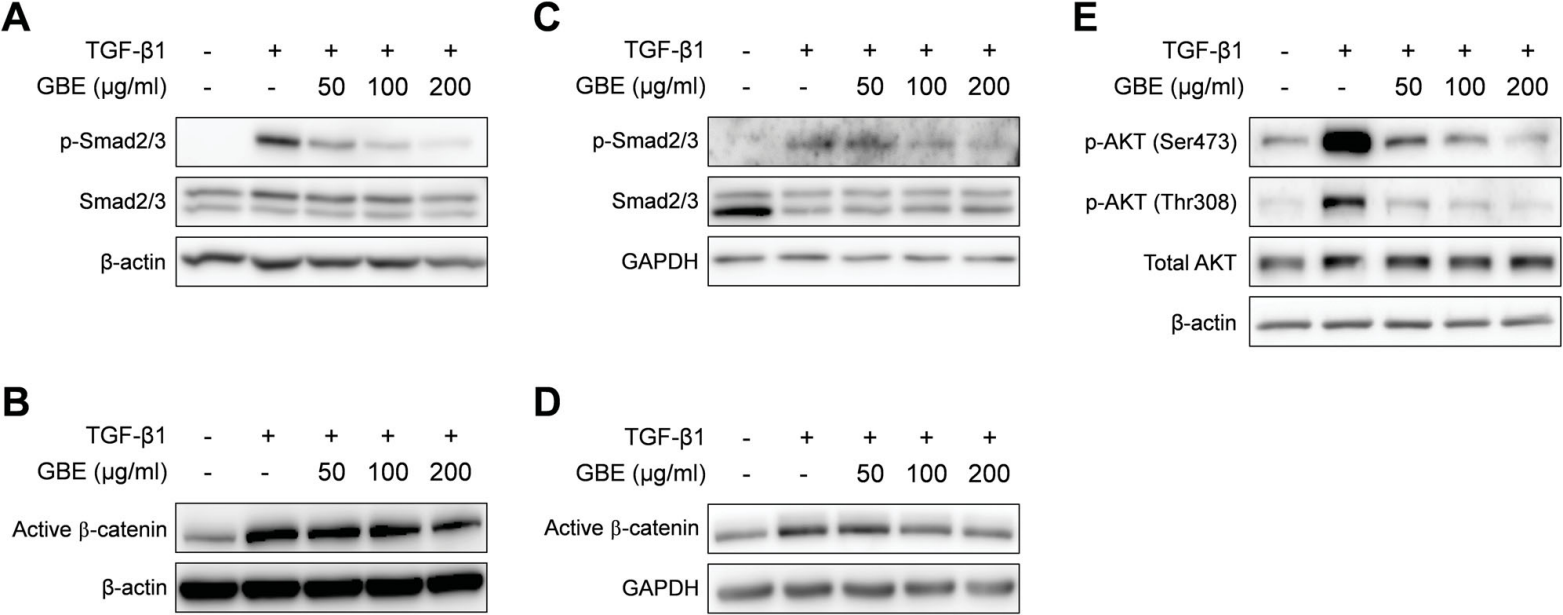
GBE inhibited TGF-β signaling in the fibroblasts. NIH3T3 cells (A and B) and normal human dermal fibroblasts (C–E) were treated with TGF-β1 (10 ng/ml) and GBE for 24 h. The protein levels of downstream targets (p-Smad2/3, active β-catenin, and p-AKT) of the TGF-β signaling pathway were analyzed by western blot. Beta-actin or GAPDH was used as a loading control.

Since GBE inhibited TGF-β-induced expression of fibrosis markers, we further investigated whether GBE modulates TGF-β signaling pathway. Smad2 and Smad3 (Smad2/3) are the key downstream transcription factors of canonical TGF-β signaling to control fibrosis. When NIH3T3 and normal human dermal fibroblasts were treated with GBE and TGF-β1, GBE significantly inhibited TGF-β-induced phosphorylation of Smad2/3 in the fibroblasts (Figure 4(A) and 4(C)). Since TGF-β can also transduce signals through Smad-independent pathways such as AKT and Wnt/β-catenin (Akhmetshina et al. 2012; Finnson et al. 2020), we examined the effects of GBE on the non-canonical pathways of TGF-β. Similar to Smad2/3, GBE inhibited TGF-β-induced activation of β-catenin and AKT phosphorylation in the fibroblasts (Figure 4(B), 4(D), and 4(E)). These results suggest that GBE ameliorates fibrosis by suppressing both the canonical and non-canonical TGF-β signaling pathways.

## Discussion

In this study, using both *in vivo* and *in vitro* models, the inhibitory effect of GBE on skin fibrosis was demonstrated by targeting both the TGF-β signaling and the transdifferentiation of adipocytes into myofibroblasts. Adipocytes have been suggested as one of the sources of myofibroblasts in SSc (Marangoni et al. 2015). Loss of intradermal adipose tissue is frequently observed in the lesional skin of patients with SSc (Marangoni et al. 2015). Moreover, the fibrous replacement of adipose tissue has consistently been observed in animal models of SSc (Marangoni et al. 2015). Treatment with TGF-β also resulted in the loss of both morphological and biochemical characteristics of adipocytes, while acquiring myofibroblast phenotypes. This transition was accompanied by a decrease in the expression of adipogenic genes and an increase in myofibroblast-associated genes (Marangoni et al. 2015).

TGF-β activates both the canonical Smad2/3 signaling pathway and non-Smad signaling pathways including MAP kinases, PI3K/AKT, c-Abl, JAK2/STAT3, and Rho-like GTPases to regulate gene expression (Finnson et al. 2020). We demonstrated that GBE suppressed the TGF-β-induced activation of Smad2/3, AKT, and β-catenin, suggesting that GBE has the potential to inhibit both canonical Smad2/3 pathway and non-canonical pathways of TGF-β signaling. Several reports suggest that the PI3K/AKT pathway plays a pathophysiological role in fibrosis (Yang et al. 2012; Liang et al. 2014; Zhang et al. 2016). AKT is activated in skin fibroblasts from patients with SSc and inhibits apoptosis by interfering with both death receptors and the mitochondrial pathway (Datta et al. 1999; Jun et al. 2005). Therefore, defective apoptosis by activated AKT observed in skin fibroblasts in SSc may contribute to the activation of fibroblasts in the skin, promoting extracellular matrix deposition (Jun et al. 2003).

TGF-β is a central mediator for both vasculopathy and fibrosis in SSc. It plays essential roles in maintaining homeostasis in both endothelial and vascular smooth muscle cells (Lafyatis 2014; Ayers et al. 2018). Several lines of evidence suggest that TGF-β plays a notable role in vasculopathy of SSc. Vascular injuries, facilitated by factors such as TGF-β and inflammatory cytokines, contribute to vascular remodeling (Ko et al. 2023). Furthermore, mesenchymal cells, originating from endothelial-to-mesenchymal transition (EndoMT) during vascular injuries, contribute to fibrosis by producing extracellular matrix components such as collagen and fibronectin (Ko et al. 2023). On the other hand, vascular injuries activate platelets, leading to the secretion of profibrotic mediators such as TGF-β and serotonin, further amplifying fibrosis in SSc (Ntelis et al. 2019; Ko et al. 2023). Therefore, TGF-β signaling may serve as a crucial link between vasculopathy and fibrosis in SSc.

Conventional immunosuppressants such as methotrexate and mycophenolate mofetil have been used in clinical practice for the treatment of fibrosis in SSc. However, their effectiveness is limited and recent clinical trials using monoclonal antibodies against IL-6 or TGF-β have not yielded favorable results (Papadimitriou et al. 2022). Therefore, a clinical unmet need remains in patients with SSc; several other compounds that target TGF-β signaling pathway are currently under investigation. GBE has been reported to exhibit various pharmacological activities such as reducing the risk of cardiovascular disease, preventing ischemia-induced oxidation, and inhibiting platelet aggregation (Tian et al. 2017). Several studies have shown that the risk of peripheral arterial disease is higher in patients with SSc due to microvascular damage and diffuse fibrosis affecting skin and internal organs (Au et al. 2011; Cannarile et al. 2015; Park et al. 2017). Therefore, considering the importance of TGF-β in vasculopathy and fibrosis of SSc and our findings demonstrating the inhibition of TGF-β signaling by GBE, it is possible that GBE may ameliorate vasculopathy in SSc, as well as skin fibrosis.

**Figure 5.**
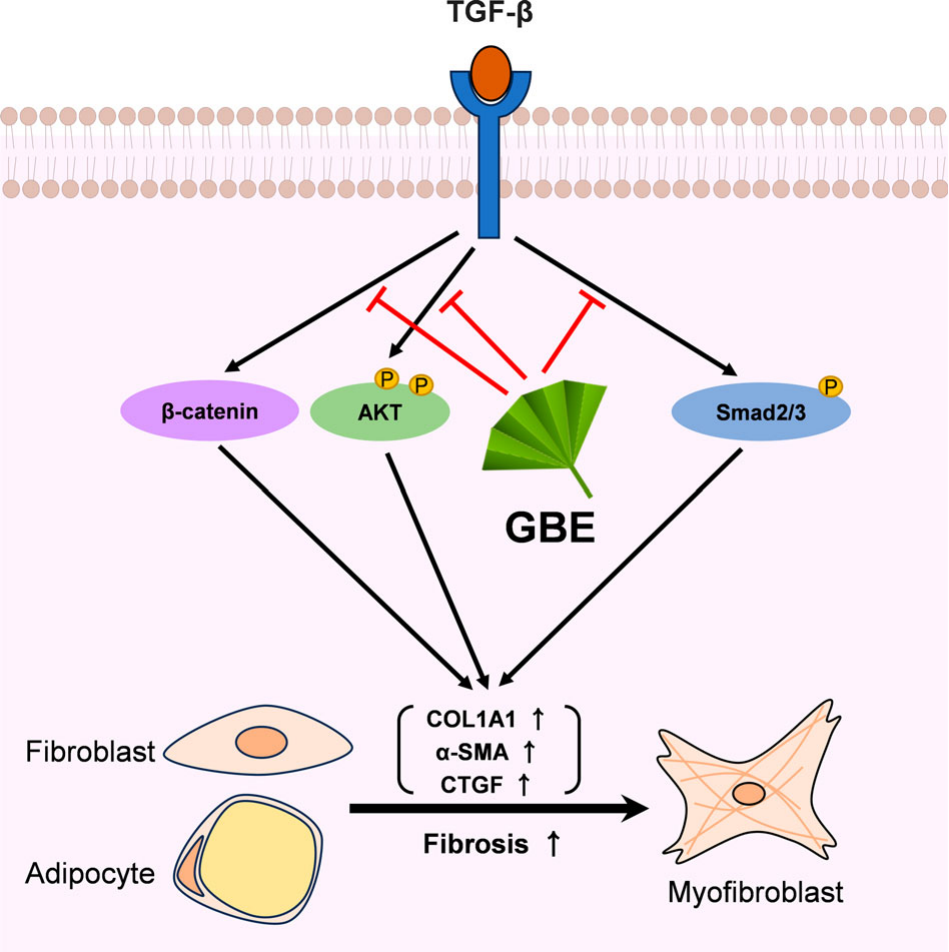
Proposed model illustrating the inhibitory effects of GBE on TGF-β downstream signaling pathways. TGF-β activates various downstream molecules, such as Smad2/3, β-catenin, and AKT, ultimately leading to fibrosis. GBE can modulate these TGF-β downstream signals, thereby inhibiting the fibrotic responses.

In conclusion, our findings demonstrate that GBE exerts anti-fibrotic effects by suppressing TGF-β-induced fibrosis through multiple mechanisms, including the modulation of signaling pathways and inhibition of the transdifferentiation of adipocytes into myofibroblasts (Figure 5). These results suggest that GBE is a promising therapeutic candidate for the treatment of SSc.

## Author contributions

Conceptualization: BL, JSR, HJ, DHS, and SGL; Acquisition of data: BL, JSR, HJ, YK, and JL; Analysis and interpretation of data: BL, JSR, HJ, YK, JL, CY, JP, DK, JL, MWS, AK, DHS, and SGL; Writing and editing of the manuscript: BL, JSR, HJ, YK, JL, DK, JL, MWS, AK, DHS, and SGL; Funding acquisition: DHS; Study supervision: DHS and SGL.
